# Global distribution and impact of knockdown resistance mutations in Aedes aegypti on pyrethroid resistance

**DOI:** 10.1186/s13071-025-06817-9

**Published:** 2025-09-24

**Authors:** Javier Tognarelli, Pablo R. Moya, Christian R. González, Ximena Collao-Ferrada

**Affiliations:** 1 Genómica UV, Escuela de Medicina, Universidad de Valparaíso, Valparaíso, Chile; 2 Departamento preclínicas, Escuela de Medicina, Universidad de Valparaíso, Valparaíso, Chile; 3 Centro de excelencia de Investigación en Medicina e Ingeniería (MEDING), Universidad de Valparaíso, Valparaíso, Chile; 4 Instituto de Fisiología, Facultad de Ciencias, Universidad de Valparaíso, Valparaíso, Chile; 5 Centro Interdisciplinario de Neurociencia de Valparaíso CINV, Universidad de Valparaíso, Valparaíso, Chile; 6 Instituto de Entomología, Facultad de Ciencias Básicas, Universidad Metropolitana de Ciencias de la Educación, Santiago, Chile

**Keywords:** *Aedes aegypti*, Pyrethroids, Insecticide resistance, Knockdown resistance, kdr, Sodium channel, VGSC, Vector control

## Abstract

The ongoing fight against mosquito-borne diseases such as dengue, Zika, and chikungunya is increasingly challenged by *Aedes aegypti* resistance to pyrethroid insecticides. This resistance is primarily driven by knockdown resistance (kdr) mutations in the voltage-gated sodium channel (*Vgsc*) gene, which have been identified in different regions with significant variability in their prevalence and impact. Managing *Ae. aegypti* populations in the face of this resistance require in-depth understanding of the global distribution of kdr mutations and their relationship to insecticide use patterns. This narrative review identifies a diverse landscape of kdr mutations, with common mutations such as V1016I, F1534C, and L982W, the latter of which has recently gained attention as an important mutation contributing to resistance in various regions, often co-occurring with other mutations and leading to enhanced resistance phenotypes. Additional mutations including V410L, G923V, S989P, I1011M/V, and D1763Y further complicate the resistance profile, indicating a dynamic evolution of resistance in response to pyrethroid use. This review also highlights significant gaps in current research, particularly in understanding the interaction between kdr mutations and other resistance mechanisms, such as metabolic detoxification by enzymes like cytochrome P450s, glutathione *S*-transferases (GST), and esterases, and the need for more standardized methodologies for data collection and reporting. The widespread use of pyrethroids has significantly contributed to the global spread of kdr mutations in *Ae. aegypti*, thereby challenging the efficacy of the current chemical vector control strategies. Despite these challenges, pyrethroids remain essential for mosquito control owing to their proven efficacy, rapid action, and versatility. However, the rising levels of resistance highlight the need for an integrated approach to vector management that combines chemical, biological, and community-based strategies. Future research should address these gaps through longitudinal studies and the development of new insecticides and resistance management strategies to address the growing threat of insecticide resistance in *Ae. aegypti* and protect public health.

**Graphical Abstract:**

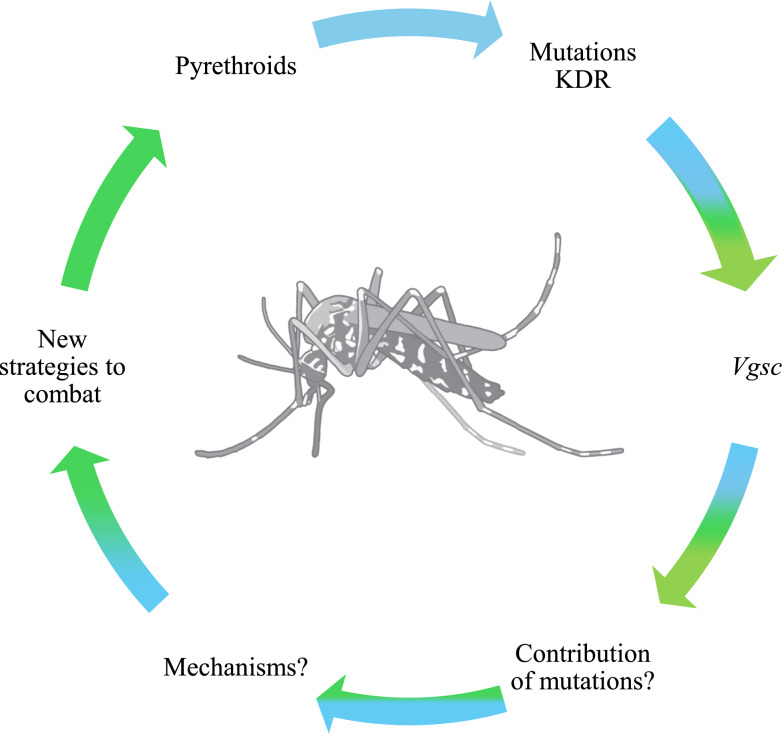

## Background

The mosquito *Aedes aegypti* (Diptera: Culicidae) is the primary vector of arboviruses, such as dengue virus (DENV), yellow fever virus (YFV), Zika virus, and chikungunya virus, particularly in urban settlements throughout tropical and subtropical areas. With an estimated 390 million annual cases of DENV infection alone, the global public health burden is significant, particularly in developing countries [1–4]. This burden has been further exacerbated by recent outbreaks, with the ongoing dengue epidemic in 2024 and early 2025 affecting multiple regions worldwide, particularly South America and Africa. Globally, over 10 million dengue cases have been reported, reflecting the alarming scale of the current crisis [5]. As of epidemiological week 2 in 2025, the Americas alone reported 134,169 suspected cases of dengue, with 37,819 laboratory-confirmed cases, 183 severe dengue cases, and 21 fatalities, reflecting a case fatality rate (CFR) of 0.016% [6]. Brazil has seen a dramatic surge, with cases increasing by 62% compared with the previous 4 weeks, while Peru reported a 38% rise. Similarly, Argentina and Paraguay continue to face significant public health challenges [6]. In Africa, Burkina Faso experienced a severe outbreak in 2023, while 15 other countries reported active DENV transmission, adding to the global burden of the disease. This surge is attributed to factors such as climate change, urbanization, and the expansion of *Ae. aegypti* habitats, compounded by the growing challenge of insecticide resistance. *The Lancet* (2024) highlights that the current outbreak reflects the urgent need for integrated vector management strategies, as traditional control methods face limitations due to the widespread emergence of knockdown resistance (kdr) mutations [5]. *Ae. aegypti* adapts well to urban environments, where it finds optimal conditions for reproduction and feeding, as it requires blood for its life cycle. It is found in densely populated urban areas as well as in rural zones [7]. With the increasing deforestation and urbanization of natural habitats and climate change due to global warming, *Ae. aegypti* have the potential to invade new habitats because of their high adaptability [7–9]. Over the past few decades, vector control strategies have relied heavily on the use of chemical insecticides, with pyrethroids emerging as the cornerstone of these efforts owing to their rapid action, efficacy, and relative safety in nontarget species. However, the widespread and prolonged use of pyrethroids has led to the development of resistance in *Ae. aegypti* populations, significantly undermining the effectiveness of these control measures [10–13].

The discovery of knockdown resistance (kdr) mutations in the voltage-gated sodium channel (*Vgsc*) gene of *Ae. aegypti* and other insects has marked a pivotal moment in the study of insecticide resistance [14, 15]. These mutations, which alter the target site of pyrethroids, reduce the binding affinity of the insecticide, allowing the mosquito to survive exposure that would otherwise be lethal [16, 17]. Since the initial identification of the L1014 F mutation in the house fly (*Musca domestica*) [18], a number of kdr mutations, including V410L, S989P, V1016I, and F1534 C, among others, have been documented in mosquito populations worldwide. The prevalence and impact of these mutations vary between regions, influenced by local insecticide use patterns and environmental factors [19–22]. Other mutations such as G923 V, L982 W, I1011M/V, D1763Y, and T1520I have also been associated to pyrethroid resistance, although their distribution may vary geographically [23–27].

Global research efforts have focused on identifying and mapping these kdr mutations to understand their distribution and to develop strategies to mitigate their impact. Studies have shown that the frequency of these mutations correlates strongly with the intensity and duration of pyrethroid use, particularly in regions with high disease transmission rates. In Latin America, Southeast Asia, and Africa, where pyrethroids are extensively used, kdr mutations have become widespread, posing a significant challenge to vector control programs [19, 28–32].

Despite significant advances in identifying and mapping kdr mutations, several critical gaps remain in our understanding of how these mutations interact with other resistance mechanisms, such as metabolic resistance, complicating efforts to develop comprehensive management strategies [33–35]. The role of detoxifying enzymes such as cytochrome P450 s, glutathione *S*-transferases (GSTs), and esterases adds further complexity as these enzymes can potentially work synergistically with kdr mutations to increase resistance levels [33, 36–40]. Additionally, the variability in the prevalence and impact of these mutations across different regions highlights the influence of local factors, which are not yet fully understood. The persistence of pyrethroids as the primary chemical control strategy, even in the face of rising resistance, underscores the urgent need for an integrated approach that balances immediate mosquito control with long-term resistance management [39, 41, 42]. This challenge calls for a detailed analysis of the global relationship between insecticide use and the prevalence of kdr mutations, particularly in regions with high disease transmission.

To ensure comprehensive coverage of relevant studies, we consulted major academic databases such as PubMed, Scopus, and Web of Science. The search primarily focused on articles published in the last 10 years, using the following keywords: ("voltage-gated sodium channel” or “sodium channel gene”) and (*Aedes* or “mosquito vector”) and (“insecticide resistance” or “vector control” or “knockdown resistance” or kdr). Additionally, we included older studies that are widely recognized and foundational in the field. Selection criteria prioritized peer-reviewed articles published in indexed journals, emphasizing relevance. We excluded articles focusing exclusively on other resistance mechanisms (e.g., metabolic resistance) unless they provided relevant insights. This approach allows for a broad yet focused analysis of the current state of knowledge on the topic.

The primary objective of this review is to analyze this relationship between global pyrethroids use and the prevalence of kdr mutations in the VGSC of *Ae. aegypti*. The review aims to highlight the presence and global distribution of these mutations and the methods used to identify them and to assess their impact on the efficacy of vector control strategies. In addition, this narrative review aims to provide a detailed examination of regional variations, with a focus on the Americas, and discusses the challenges and future directions in managing insecticide resistance in *Ae. aegypti*.

## Vector control strategies

Vector control strategies are essential to prevent and reduce arbovirus infections and mitigate their impact on health systems. A combined strategy that incorporates several measures may increase overall efficiency. Current strategies to combat mosquitoes include community engagement and education, as well as physical, biological, and chemical control, where evidence suggests integrated interventions are more effective [9, 43, 44]. Community education and awareness are essential to achieve population participation in the elimination of breeding sites and other prevention and control measures. The involvement of communities in the management of mosquitoes has demonstrated significant success in reducing the spread of diseases [44]. Despite historical effectiveness of community-driven mosquito control initiatives, they face challenges in sustainability and resource consumption [9].

Physical control strategies aim to reduce mosquito populations by targeting and eliminating potential breeding sites. Female *Ae. aegypti* deposit their eggs on damp surfaces just above the waterline, such as the inner surfaces of discarded containers (abandoned tires, containers, receptacles, and small water bodies). To effectively manage mosquito populations and reduce the transmission of vector-borne diseases, various control measures have been implemented, ranging from environmental management to biological control—which involves the introduction of organisms that infect, prey on, parasitize, or compete with target species—and advanced biocontrol techniques [45, 46]. A summary of these nonchemical strategies along with selected evidence is presented in Table 1.

**Table 1:** Summary of strategies for vector mosquito control and population reduction

Control measure	Description	References
Elimination of stagnant water	Remove objects or areas where stagnant water might collect, ensure containers such as pots and water barrels are either covered or emptied to prevent mosquito breeding	[2, 49]
Mosquito nets and physical barriers	Use mosquito nets for daytime sleep, install screens on doors and windows, and wear protective clothing that covers arms and legs to reduce exposure to mosquitoes	[2–4]
Mosquito repellents	Spatial repellents containing transfluthrin have shown a 34.1% reduction in the transmission rates of *Aedes*-borne viruses, such as dengue and Zika, in a trial conducted in Iquitos, Peru	[50]
Essential oils	Effective as larvicides and repellents, serving as a feasible alternative to synthetic repellents	[51–53]
RNAi-based larvicides	Development of an RNAi-based microalgal larvicide that induces significant damage to the integumentary structure and midguts of larvae, leading to increased mortality	[54]
Sterile insect technique (SIT)	Sterilization of a male population using gamma radiation, with up to 99% sterility depending on the radiation dose	[55, 56]
Incompatible insect technique (IIT)	Use of *Wolbachia pipientis* to sterilize males. Releasing large numbers of sterilized males into a mosquito population leads to no offspring and eventual population collapse	[56]
Ovitraps and larvitraps	Ovitraps and larvitraps are used to monitor and control mosquito populations, aiding surveillance and sometimes incorporating insecticides for population reduction. Ovitraps containing larvicide pyriproxyfen have demonstrated the ability to inhibit the emergence of adult mosquitoes for over 30 weeks	[57, ]
Bacterial agents	Use of bacteria such as *Bacillus thuringiensis* var. *israelensis* (Bti), which produces toxin proteins with larvicidal effects	[59, 60]
Entomopathogenic fungi	Use of *Metarhizium anisopliae* and *M. brunneum* against *Ae. aegypti* larvae. Larvicidal and adulticidal activity of *Aspergillus tamarii*, *Beauveria bassiana*, and *Trichoderma longibrachiatum*, among others, showing promising results in reducing mosquito populations	[61–64]
Larvivorous fish	Species such as *Gambusia affinis*, *G. holbrooki*, and *Poecilia reticulata* significantly decrease mosquito larvae in water containers. However, their introduction into non-native ecosystems raises concerns about potential impacts on local biodiversity, emphasizing the need for careful management	[65, 66]

### Chemical vector control strategies

Chemical control involves strategic use of insecticides to manage mosquito populations, particularly in regions of high disease transmission. Several classes of insecticides are used to control *Ae. aegypti* populations, each acting through different mechanisms to disrupt the insect’s nervous system, ultimately leading to paralysis and death [43, 47, 48]. The major classes of insecticides include pyrethroids, organochlorines, organophosphates, and carbamates. Organophosphates and carbamates work by inhibiting acetylcholinesterase, an enzyme critical to nerve function, thereby preventing the breakdown of acetylcholine and causing continuous nerve stimulation. In contrast, pyrethroids and organochlorines target the voltage-gated sodium channel (VGSC), binding to the channel and preventing its proper deactivation and inactivation. This disrupts the electrical signaling in the nervous system of insects, resulting in fatal neurological failure [49–51].

Among insecticidal compounds, pyrethroids are synthetic analogs of pyrethrins, which are naturally derived from the flowers of the chrysanthemum plant (*Tanacetum cinerariaefolium*) [52]. Commonly used pyrethroids include permethrin, deltamethrin, and cypermethrin, which are widely employed in vector control programs, and they have been extensively used for over three decades owing to their rapid action and relatively low toxicity to mammals, making them a cornerstone of chemical mosquito control strategies worldwide. Nevertheless, classifying pyrethroids as showing “low toxicity to mammals” requires nuance. While mammalian sodium channels exhibit lower sensitivity to pyrethroids compared with insects, and metabolic detoxification is generally more efficient, these compounds can still pose risks, particularly for vulnerable populations such as neonates and individuals with impaired detoxification pathways. Moreover, pyrethroids have been shown to interact with voltage-gated calcium and chloride channels, suggesting potential neurotoxic effects beyond sodium channel modulation [17]. Additional concerns arise from pyrethroid metabolites, which may exhibit greater endocrine-disrupting potential than their parent compounds. Furthermore, photodegradation byproducts, such as dibenzofurans, have been identified as environmentally persistent and potentially harmful to both wildlife and humans [53]. Thus, while pyrethroids are considered safer than many other insecticides, their toxicity should be assessed within a broader context that considers exposure levels, bioaccumulation, and long-term effects.

Despite these concerns, the ability of pyrethroids to rapidly incapacitate mosquitoes and agricultural pests has solidified their role as a primary tool in chemical control strategies [9, 17, 53, 54]. In addition to their use in indoor residual spraying (IRS), pyrethroids are the only insecticides approved for treating insecticide-treated nets (ITNs) and long-lasting insecticidal nets (LLINs), which have proven to be essential tools in reducing the transmission of vector-borne diseases, including those caused by *Ae. aegypti* [52]. Recommendations from the World Health Organization (WHO) have highlighted the need for improved LLINs with enhanced durability and sustainability to counteract growing pyrethroid resistance [55]. Additionally, ongoing research suggests that innovations in LLIN materials and insecticide formulations could extend their lifespan, increase cost-effectiveness, and reduce environmental impact [56].

However, the very success of pyrethroids in controlling mosquito populations has also contributed to their downfall as widespread use has exerted intense selection pressure, leading to the rapid development of resistance mechanisms within *Ae. aegypti* populations. Consequently, research efforts have focused on identifying the genetic and biochemical changes responsible for resistance. It was during this period that the concept of knockdown resistance (kdr) emerged as a major focus of study, with the discovery of kdr mutations representing a breakthrough in understanding the molecular basis of pyrethroid resistance [16, 52, 57, 58].

## Structural and functional overview of the *Vgsc* gene in *Aedes aegypti*

The voltage-gated sodium channel (VGSC) protein represents a pivotal element of the nervous system in insects, exerting a fundamental role in the generation and propagation of action potentials in nerve cells. The VGSC, which is encoded by the *Vgsc* gene, is a transmembrane protein complex that allows sodium ions to flow into neurons in response to changes in membrane potential. This facilitates the rapid depolarization that is necessary for the transmission of nerve impulses [59].

### Structural features of the VGSC protein

The VGSC protein in *Ae. aegypti*, like that of other insects, is composed of four homologous domains (I–IV), each consisting of six transmembrane segments (S1–S6). The S4 segment in each domain functions as the voltage sensor, detecting changes in membrane potential and triggering conformational changes that regulate the opening or closing of the channel. The S5 and S6 segments constitute the actual pore through which sodium ions are permitted to pass. The high degree of conservation observed across species for these domains highlights their essential role in maintaining the proper functioning of the nervous system [60, 61].

The opening and closing of the channel are tightly regulated processes that are essential for normal neuronal activity. As the membrane potential becomes more positive, the S4 segments move outward, thereby opening the channel and allowing sodium ions to enter the cell. This influx of sodium ions results in a depolarization of the membrane potential, which propagates the action potential along the neuron. Once the membrane potential has been restored to its resting state, the channel closes, thereby halting the influx of sodium and resetting the neuron for subsequent signaling. The importance of these structural elements was further highlighted by studies showing that mutations in specific regions of the VGSC protein, particularly in the S6 segment of domain II, could confer resistance to pyrethroids and dichlorodiphenyltrichloroethane (DDT). These insecticides act by binding to the VGSC and disrupting its normal function, resulting in prolonged opening of the sodium channels. This results in sustained depolarization of the neuron, preventing the proper transmission of nerve impulses and ultimately causing paralysis and death in the mosquito. The efficacy of pyrethroids in mosquito control is largely due to their ability to target this critical component of the mosquito nervous system [16, 50, 60–65].

### Understanding VGSC and resistance mechanisms

**Fig. 1:**
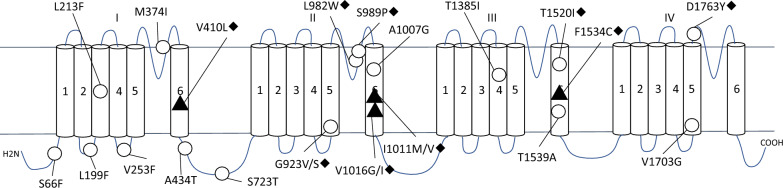
Mutations in the voltage-gated sodium channel (VGSC) associated with pyrethroid resistance in *Aedes aegypti*. The diagram shows key amino acid substitutions in the VGSC of *A. aegypti*. Black indicates previously known mutations, red highlights those with proven contributions to resistance from multiple electrophysiological studies (e.g., I1011M, V1016G, and F1534 C), and blue marks novel mutations identified in the study of Kasai et al. [69]. Adapted from Kasai et al., Science Advances, © 2022 The Authors, published by the American Association for the Advancement of Science under a Creative Commons Attribution NonCommercial License 4.0 (CC BY-NC 4.0)

As the importance of the *Vgsc* gene in insecticide action became clear, research began to focus on understanding how mutations in this gene could lead to resistance. To date, at least 15 nonsynonymous mutations in VGSC associated with varying levels of resistance to pyrethroids have been identified. Among these, the most reported and impactful mutations are V410L, S989P, V1016G/I, F1534 C, and D1763Y, particularly when they co-occur, leading to a synergistic effect that significantly enhances resistance [59, 66–68]. Studies have shown that specific point mutations in the *Vgsc* gene can alter the structure of the sodium channel, reducing the binding affinity of pyrethroids and thereby diminishing their efficacy [65]. To facilitate a comprehensive understanding of the kdr mutations described in Sects. “The L1014 F mutation and its impact on VGSC function” through “Additional kdr variants contributing to pyrethroid resistance”, Fig. 1 illustrates these key mutations in the voltage-gated sodium channel (VGSC), including novel substitutions identified in recent studies [69]. This visual representation complements Table 2, which provides a detailed summary of amino acid substitutions, VGSC location, resistance mechanism, and geographic distribution of major mutations. While Fig. 1 includes newly discovered mutations, the functional characterization of most well-established kdr mutations is thoroughly discussed in Dong [16].

**Table 2:** Geographic distribution and resistance mechanisms of kdr mutations in the voltage-gated sodium channel (VGSC) of *Aedes aegypti*

Mutation	Amino acid substitution	VGSC location	Resistance mechanism and impact	Geographic distribution	Resistance level	Interaction type	References
V410L	Valine (V) to leucine (L)	S1–S2 linker of domain I	Alters channel conformation, contributing to resistance with V1016I	Mexico, Colombia	Moderate alone, high with V1016I	Synergistic with V1016I	[83, 93]
G923 V	Glycine (G) to valine (V)	Segment 5 of domain II (IIS5)	Associated with moderate resistance due to altered channel sensitivity	Asia, Latin America	Moderate	Synergistic with F1534 C	[47]
L982 W	Leucine (L) to tryptophan (W)	Segment 5 of domain II (IIS5)	Contributes to resistance by altering channel structure, reducing insecticide sensitivity	South Vietnam, Asia	High	Synergistic with F1534 C	[47, 86]
S989P	Serine (S) to proline (P)	Near V1016I in S6 of domain II	Increases VGSC stability, reducing efficacy of pyrethroids with V1016I	India, Myanmar, Nigeria	High with V1016G	Synergistic with V1016G	[47]
I1011M	Isoleucine (I) to methionine (M)	Segment 6 of domain II (IIS6)	Enhances resistance by altering ionic flow through VGSC	Latin America	High with F1534 C	Synergistic with V1016I, F1534 C	[27]
I1011 V	Isoleucine (I) to valine (V)	Segment 6 of domain II (IIS6)	Enhances resistance with V1016I, F1534 C	Southeast Asia, Latin America	High with F1534 C	Synergistic with F1534 C	[32]
V1016G	Valine (V) to glycine (G)	Segment 6 of domain II (IIS6)	Stabilizes channel structure, high resistance with F1534 C	Thailand, Myanmar	High with F1534 C	Synergistic with F1534 C	[74]
V1016I	Valine (V) to isoleucine (I)	S6 in domain II of VGSC	Reduces pyrethroid binding affinity, allowing channel survival; prevents the channel from remaining open	Colombian Caribbean, Costa Rica, Mexico, Venezuela	High alone, very high with F1534 C	Synergistic with F1534 C	[19, 32]
T1520I	Threonine (T) to isoleucine (I)	Segment 6 of domain III (IIIS6)	Associated with localized resistance due to specific selection pressures	India	Moderate with F1534 C	Synergistic with F1534 C	[23]
F1534 C	Phenylalanine (F) to cysteine (C)	S6 in domain III of VGSC	Confers moderate resistance alone, high with V1016I; increases channel closure rate, reducing pyrethroid efficacy	India, Thailand, Brazil, Venezuela, Costa Rica	Moderate alone, high with V1016I	Synergistic with V1016I	[10, 32]
D1763Y	Aspartate (D) to tyrosine (Y)	Segment 6 of domain IV (IVS6)	Minor resistance effect alone; requires further studies to assess interaction with other kdr mutations; found in combination with V1016G, S989P, and F1534 C, suggesting potential synergistic effects	Taiwan	Low alone, potential increase with V1016G	Synergistic with V1016G, requires further studies	[66, 84]

#### The L1014 F mutation and its impact on VGSC function

One of the earliest and best known kdr mutations is L1014 F, which involves the substitution of a leucine residue with phenylalanine in the S6 segment of domain II (IIS6) of the VGSC protein. This mutation was first identified in the housefly (*M. domestica*) and was later found in several other insect species [18, 30, 69], with the exception of *Ae. aegypti*. The L1014 F mutation, like other kdr mutations, alters the binding site of pyrethroids to the sodium channel, reducing their ability to keep the sodium channel open. As a result, the mutant VGSC can close even in the presence of the insecticide, allowing the neuron to return to its resting state and enabling the mosquito to survive exposure to the chemical. This discovery was critical because it provided the first concrete evidence of a genetic basis for pyrethroid resistance in mosquitoes. In *Ae. aegypti*, codon constraints prevent the L1014 F mutation, requiring two mutational events within the same codon to induce this specific mutation. Therefore, it has not been observed in these mosquitoes, and it is unlikely to occur [30, 70].

#### V1016G and F1534 C mutations: synergistic effects on resistance

Subsequent research identified additional kdr mutations, including V1016G and F1534 C, which were shown to contribute to varying degrees of resistance across different insect populations. The identification of these mutations has been critical in elucidating the underlying mechanisms of insecticide resistance, particularly in vector species such as *Ae. aegypti*. While both mutations can appear independently and confer high levels of resistance, their combination often leads to an even stronger resistance phenotype [59, 66–68].

The V1016G/I mutation is one of the most extensively reported kdr mutations in *Ae. aegypti*. This mutation occurs in the S6 segment of domain II (IIS6) of the VGSC protein and involves the substitution of valine (V) with either glycine (G) or isoleucine (I) at position 1016 [66, 67, 71]. The V1016G/I mutation is particularly prevalent in *Ae. aegypti* populations in Latin America and Southeast Asia, where pyrethroid resistance has become a significant challenge [71, 72]. Studies have shown that the presence of V1016G correlates with high levels of resistance to pyrethroids, particularly in areas where these insecticides have been heavily used over extended periods [31, 72].

The F1534 C mutation is also one of the most extensively reported and frequent kdr mutations in *Ae. aegypti*, located in the segment S6 of domain III (IIIS6) of the VGSC, involves the substitution of phenylalanine (F) with cysteine (C) at position 1534. This mutation has been widely reported in *Ae. aegypti* populations across Asia, Africa, and Latin America, and it is considered one of the most prevalent kdr mutations globally owing to its high frequency in multiple geographic regions and its role in pyrethroid resistance [10].

While the F1534 C mutation alone confers several-fold resistance, its combination with V1016G significantly increases the resistance phenotype, making it particularly difficult to control mosquito populations in regions where both mutations are prevalent [27, 31, 72–75]. This mutation is associated with a decrease in the efficacy of pyrethroid-based interventions, leading to the persistence of mosquito populations despite control efforts.

#### S989P and V410L mutations

The S989P mutation, located near the V1016G/I mutation in the S6 segment of domain II (IIS6), involves the substitution of serine (S) for proline (P) at position 989. Although less detected than V1016G/I or F1534 C, the S989P mutation has been identified in several *Ae. aegypti* populations, particularly in association with V1016G/I. This proximity suggests that S989P may act as a compensatory mutation, enhancing the overall resistance phenotype when present alongside V1016G/I. Research suggests that the S989P mutation contributes to the stability of the V1016G/I protein structure, further reducing the binding efficacy of pyrethroids [34, 57, 67]. The combination of S989P and V1016G/I is of particular concern as it has been associated with high levels of resistance in regions where pyrethroids are used extensively, and their frequent co-occurrence suggests they may be subject to similar selective pressures. Further phylogenetic and population genetics studies are needed to clarify their evolutionary relationship.

The V410L mutation is one of the most recent additions to the catalog of kdr mutations known in *Ae. aegypti*. This mutation occurs in the S1–S2 linker of domain I (IS6) of the VGSC protein, where valine (V) is replaced by leucine (L) at position 410. The V410L mutation has been identified primarily in populations from Africa and South America and is thought to contribute to pyrethroid resistance by altering the channel’s conformational state, affecting the gating mechanism of the VGSC [76–78]. Studies suggest that the V410L mutation, while less common than mutations such as V1016I or F1534 C, plays a significant role in resistance when present alongside other kdr mutations. For instance, mosquitoes carrying both V410L and V1016I mutations have shown enhanced resistance to pyrethroids compared with those with only a single mutation [11, 20, 77]. The exact mechanism by which V410L contributes to resistance is still under investigation, but it is believed to influence the interaction between the VGSC and pyrethroids by altering the protein’s structural flexibility.

#### Additional kdr variants contributing to pyrethroid resistance

Beyond the commonly studied kdr mutations such as S989P, V410L, V1016G/I, and F1534 C, recent research has identified other mutations in *Ae. aegypti* that contribute to a more complex pattern of pyrethroid resistance. These additional mutations include G923 V and L982 W in domain II, segment 5 (IIS5), which are linked to moderate resistance levels in specific populations owing to their impact on channel sensitivity. Furthermore, I1011M and I1011 V, located in domain II, segment 6 (IIS6) often co-occur with other kdr mutations, enhancing resistance by altering ion flow through the sodium channel [15, 30, 68, 75, 79–81]. Mutations such as D1763Y in domain IV, segment 6 (IVS6) and T1520I in IIIS6 are less prevalent and tend to reflect localized selection pressures, adding further diversity to the genetic profile of resistance [23, 49]. These variants, unlike the more common mutations, often exhibit region-specific patterns and contribute unique mechanisms to the overall resistance phenotype, making their study essential for understanding and managing resistance in different ecological settings. This highlights the need for region-specific approaches and diagnostic improvements to address the multifactorial nature of resistance in *Ae. aegypti* [23, 27, 82].

These studies highlighted that kdr mutations could occur in combination, leading to synergistic effects that enhance resistance levels beyond what might be expected from single mutations alone.

### Evolution and spread of kdr mutations

The spread of kdr mutations in *Ae. aegypti* populations is a testament to the adaptive capacity of mosquitoes under intense selection pressure. As pyrethroids continue to be used extensively in vector control programs, the frequency of kdr mutations increased in many regions. Studies from Latin America, Southeast Asia, and Africa have documented the increasing prevalence of these mutations, often correlating their spread with the intensity and duration of pyrethroid use [30, 31, 83].

Research has also shown that kdr mutations do not occur in isolation, but are often present in combination with other mutations or resistance mechanisms, resulting in enhanced resistance phenotypes. This combinatorial effect of multiple kdr mutations, along with other resistance mechanisms such as metabolic detoxification, has made managing pyrethroid resistance increasingly challenging. The impact of detoxifying enzymes, such as cytochrome P450 s, glutathione *S*-transferases (GSTs), and esterases, is particularly important and requires further investigation, as these enzymes can act in concert with kdr mutations to dramatically increase resistance levels [33, 35, 84–86].

## Global research efforts to identify and map kdr mutations in *Ae. aegypti* populations

The identification and mapping of knockdown resistance (kdr) mutations in *Ae. aegypti* have been central to global research efforts, driven by the critical need to address the increasing challenge of pyrethroid resistance. These mutations in the *Vgsc* gene are key contributors to the reduced efficacy of pyrethroid-based insecticides, which are widely used in mosquito control programs.

### Initial studies and mapping efforts

**Fig. 2:**
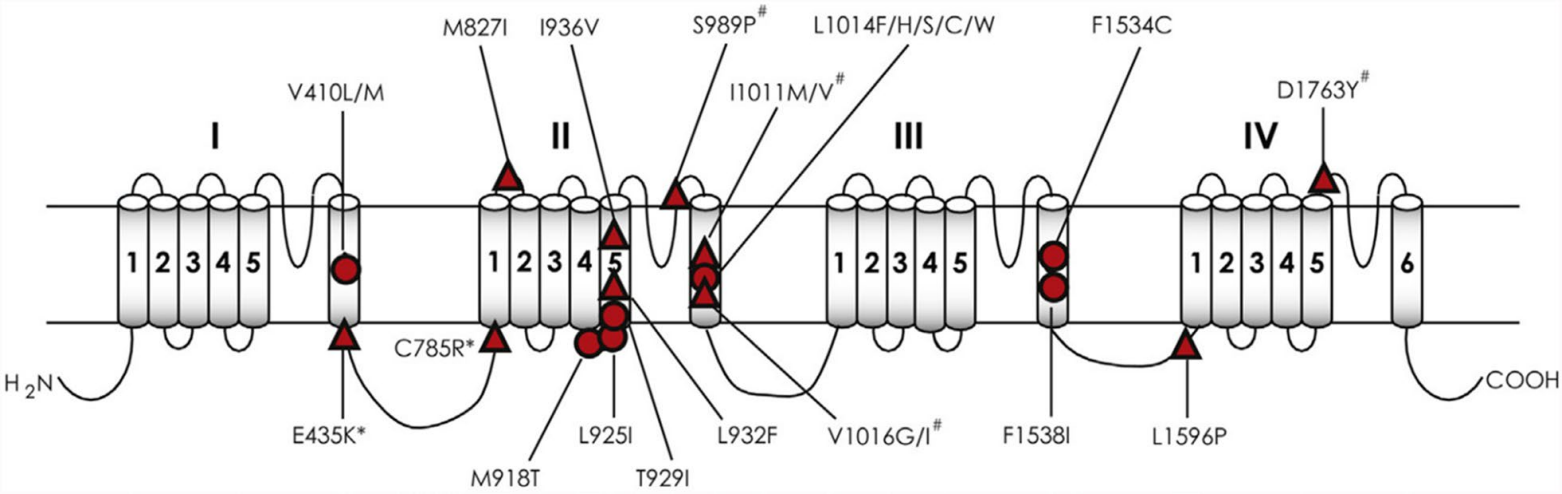
Geographic distribution of mutations in the *Vgsc* gene associated with pyrethroid resistance in *Ae. aegypti*. The shaded areas represent the three main study regions: Africa (light-yellow circle), Latin America-Caribbean (light-blue circle), and Southeast Asia (light-green circle). Mutations are color-coded according to their location within the VGSC domains: domain I (red): V410L; domain II (magenta): G923 V, L982 W, S989P, I1011M/V, and V1016G/I; domain III (green): T1520I and F1534 C; domain IV (blue): D1763Y

The discovery of kdr mutations began with the identification of specific point mutations in the *Vgsc* gene that confer resistance to pyrethroids. Initial studies focused on Latin American populations of *Ae. aegypti*, where V1016I and F1534 C were identified as significant drivers of resistance, laying the groundwork for broader surveys across regions [19, 66, 72, 82]. Subsequent surveys in Southeast Asia and Africa revealed a similarly widespread distribution of these mutations, underscoring the global scope of resistance and the need for coordinated international surveillance efforts [20, 31, 67]. Figure 2 shows a color-coded map illustrating the major VGSC mutations frequently detected across key geographic regions, highlighting local adaptations of *Ae. aegypti* populations to regional insecticide pressure.

### Regional variations and global patterns

The prevalence of kdr mutations shows significant variability across different geographical regions, influenced by local insecticide usage patterns, mosquito population dynamics, and environmental factors.

#### Latin America

In Latin America, particularly in Brazil, Mexico, and the Caribbean, the V1016I and F1534 C mutations are highly prevalent. These mutations have been found at high frequencies in *Ae. aegypti* populations, correlating with the extensive use of pyrethroids in urban vector control programs. The co-occurrence of V1016I and F1534 C is particularly problematic in these regions, as it leads to higher resistance levels and reduces the effectiveness of control measures. The presence of the V410L mutation in certain areas further complicates the resistance profile, highlighting the significant selection pressure exerted by pyrethroid use and the challenges faced by vector control programs in this region [22, 72, 77, 83].

#### Southeast Asia

Research in Southeast Asia has revealed a more complex resistance landscape, with multiple kdr mutations occurring within the same populations. The V1016G mutation, which is more common than V1016I in this region, often co-occurs with F1534 C. This combination has been documented in countries such as Vietnam and Thailand, where pyrethroid resistance has become a critical problem, often compounded by other resistance mechanisms such as metabolic detoxification. This combinatorial resistance poses a significant challenge to the continued efficacy of pyrethroids in this region, prompting efforts to explore alternative control strategies, such as the use of nonpyrethroid insecticides and integrated vector management approaches [10, 24, 35, 47, 57, 69].

#### Africa

Africa presents a heterogeneous landscape regarding the prevalence of kdr mutations in *Ae. aegypti* populations. In West Africa, particularly in countries such as Ghana, Côte d'Ivoire, and Nigeria, the V410L, V1016I, and F1534 C mutations have been documented, with different frequencies across regions [87, 88]. While these mutations are less common in some parts of Africa compared with other regions, they are strongly associated with significant resistance to pyrethroids in the areas where they occur, thereby complicating mosquito control efforts in regions with high transmission rates of mosquito-borne diseases. The recent identification of V410L, V1016I, and F1534 C in Niger [78], and S989P in Nigeria [89], further underscores the evolving and complex nature of insecticide resistance across Africa and highlights the need for targeted surveillance and adaptive resistance management strategies [20, 29, 31, 73].

### Impact of kdr mutations on resistance phenotypes

The presence of kdr mutations in *Ae. aegypti* populations significantly impacts the phenotypic expression of resistance, leading to higher survival rates following insecticide exposure. The co-occurrence of different kdr mutations often results in varying levels of resistance, complicating predictions and management strategies. For instance, the combination of V1016I and F1534 C mutations has been shown to confer higher resistance than either mutation alone, sometimes several hundreds or thousands of times higher, underscoring the complexity of resistance mechanisms in *Ae. aegypti* [10, 34, 69, 77, 90, 91]. While substantial progress has been made in mapping kdr mutations globally, challenges remain, particularly in the variability of detection methods and the need for comprehensive surveillance programs that include both rural and urban settings.

## Focus on the Americas: in-depth analysis of kdr mutations in *Aedes aegypti* populations

The Americas have been central to the study of kdr mutations in *Ae. aegypti*, largely owing to the extensive and long-term use of pyrethroid insecticides for vector control. This section provides a detailed analysis of the prevalence and impact of kdr mutations across North, Central, and South America, and explores the correlation between mutation frequency and insecticide use patterns.

### North America

In North America, particularly in the USA and Mexico, kdr mutations have been extensively studied owing to their significant public health implications, especially concerning diseases such as dengue, Zika, and West Nile virus. The V1016I and F1534 C mutations are the most commonly reported in these populations [83, 92, 93].

#### USA

In the USA, particularly in southern states such as Florida and Texas, the frequency of V1016I and F1534 C mutations has been documented in several studies. Additionally, V410L has been identified in certain areas, further complicating resistance management efforts [21, 92, 94]. These mutations have been linked to the widespread use of pyrethroids in both agricultural and urban settings, where they are applied extensively for mosquito control. The correlation between these mutations and pyrethroid use suggests that high selective pressure has driven the evolution and spread of these resistance alleles. However, while these mutations are of concern, resistance levels in the USA are not yet as high as in some Latin American countries, possibly owing to differences in the intensity of insecticide use and the implementation of integrated vector management strategies [21, 22, 93].

#### Mexico

In Mexico, the prevalence of kdr mutations is significantly higher, especially in urban areas with intensive insecticide use. The V1016I and F1534 C mutations are common in *Ae. aegypti* populations, with studies suggesting that these mutations are nearly fixed in certain regions. This high prevalence correlates strongly with the extensive use of pyrethroids in public health campaigns to control dengue outbreaks. The near-fixation of these mutations in some areas suggests that resistance management strategies may need to be revised to prevent further escalation of resistance levels [22, 66, 95–97].

### Central America

Central America presents a diverse landscape regarding kdr mutation prevalence, influenced by varying levels of insecticide use across different countries. In countries such as Costa Rica and Panama, the V1016I and F1534 C mutations are prevalent, though their frequency varies widely [19, 30].

#### Regional variability in Central America

The variability in mutation frequency across Central America can be attributed to multiple factors, including differences in public health infrastructure, the scale of insecticide use, and broader socioeconomic and environmental conditions [30]. For instance, in countries with more robust vector control programs, such as Costa Rica, there is evidence of a more moderated rise in mutation frequencies, possibly due to the implementation of integrated vector management approaches that reduce reliance on chemical controls alone [19]. However, beyond vector control strategies, structural determinants such as access to sanitation, reliable water supply, waste management, and socioeconomic disparities may also play a role in shaping the selective pressures on mosquito populations. In regions where these conditions are suboptimal, inconsistent insecticide use, often reactive to dengue outbreaks, may further contribute to the selection of resistant populations [19, 30].

### South America

South America has been one of the regions most affected by the spread of kdr mutations, particularly in Brazil, where pyrethroid use has been extensive and sustained over many years [98]. This has resulted in some of the highest recorded frequencies of kdr mutations worldwide [99].

#### Brazil

Brazil stands out as a hotspot for the emergence of kdr mutations, with studies consistently reporting high prevalence of both V1016I and F1534 C mutations [99]. The widespread use of pyrethroids in both agricultural and urban settings has exerted significant selective pressure on *Ae. aegypti* populations, resulting in the rapid spread of these resistance alleles [98, 100]. The situation in Brazil is further complicated by the presence of other resistance mechanisms, such as metabolic resistance, which, when combined with kdr mutations, pose a formidable challenge for vector control. The high levels of resistance observed in Brazil underscore the need for a comprehensive approach to resistance management, including the development of new insecticides and the implementation of nonchemical control methods [101].

#### Other South American countries

A similarly concerning situation is observed in other South American countries, including Colombia, Peru, and Argentina, though with some regional variations. In Colombia, the prevalence of kdr mutations is high in both rural and urban areas, which correlates with the widespread and often indiscriminate use of pyrethroids [72]. Evidence from Peru indicates the presence of regional differences, with some areas exhibiting elevated frequencies of resistance mutations. This is likely attributable to localized disparities in insecticide use [102]. Argentina, while also experiencing significant resistance, has seen some success in managing resistance through integrated vector management strategies that combine chemical and nonchemical controls [28, 48].

## Impact of global insecticide use on kdr mutation prevalence in *Ae. aegypti*

The prevalence of kdr mutations in *Ae. aegypti* is strongly associated with insecticide application patterns, particularly with pyrethroids such as permethrin, deltamethrin, and lambda-cyhalothrin [76]. Regional variations in application intensity, method (e.g., indoor residual spraying, insecticide-treated nets, and space spraying), and specific pyrethroid types contribute to differing selection pressures [88]. Recognizing these correlations is essential to refining vector control strategies and adapting to local resistance patterns effectively [93].

### Regional correlations and variation

The prevalence of kdr mutations in *Ae. aegypti* varies significantly across regions, influenced by local insecticide application practices, environmental conditions, and mosquito population dynamics [72]. In regions such as Latin America and Southeast Asia, where pyrethroids, and other insecticides, are predominantly used in both agriculture and vector control [17, 103], kdr mutations such as V1016I, F1534 C, and V410L have reached high frequencies [104]. These regional differences underscore the role of specific insecticide practices in driving selection pressures, resulting in varying levels of resistance across *Ae. aegypti* populations worldwide [30].

#### Latin America

In Latin America, pyrethroids such as permethrin, deltamethrin, and lambda-cyhalothrin are widely used in mosquito control programs. In countries such as Brazil and Mexico, these insecticides are a mainstay of urban vector control strategies aimed at reducing the incidence of dengue, Zika, and chikungunya [93]. The high selective pressure of frequent use of these pyrethroids has led to the rapid spread of the V1016I and F1534 C mutations, resulting in significant resistance problems [76]. In some regions of Brazil, these mutations are nearly fixed in the *Ae. aegypti* populations, complicating efforts to control mosquito-borne diseases.

#### Southeast Asia

In Southeast Asia, the use of pyrethroids such as permethrin and deltamethrin is widespread, particularly in countries such as Thailand, Malaysia, and Vietnam. These pyrethroids are commonly applied through space spraying and treated bed nets as part of large-scale vector control campaigns [105]. The consistent and widespread use of pyrethroids in Southeast Asia region has led to a high prevalence of kdr mutations, particularly V1016G and F1534 C [47, 90]. The use of permethrin-treated bed nets has also contributed to the selection pressure in these regions, further entrenching resistance in local mosquito populations [35].

#### Africa

In Africa, insecticides show regional variability, impacting the prevalence and distribution of kdr mutations. Pyrethroids, including deltamethrin and permethrin, are extensively utilized in indoor residual spraying (IRS) and long-lasting insecticidal nets (LLINs) across much of the continent [106]. In West Africa, particularly in areas where deltamethrin-treated nets are frequently deployed, mutations such as V1016I, F1534 C, and V410L have been reported, with a notable rise in mutation frequencies linked to consistent pyrethroid exposure [88]. Conversely, regions where pyrethroid usage is less prevalent or where alternative insecticides are integrated into vector control strategies tend to show lower frequencies of these mutations. This heterogeneous distribution underscores the need for tailored resistance management strategies that consider local insecticide practices and environmental conditions [87].

### Fitness costs and the stability of kdr mutations

The persistence of kdr mutations within *Ae. aegypti* populations is shaped by a balance between selection pressure exerted by insecticide exposure and fitness costs associated with resistance alleles. Smith et al. demonstrated that both kdr mutations and cytochrome P450 (CYP)-mediated detoxification impose fitness costs, though they affect different physiological traits. CYP-based resistance significantly reduces longevity and mating competitiveness, whereas kdr mutations exhibit a fitness cost, though the specific biological function affected remains unclear [107]. Similarly, Silva et al. showed that mosquitoes carrying the 410L + 1016I + 1534 C haplotype experience higher fitness costs compared with those with only the 1534 C allele. However, when exposed to deltamethrin, the 410L + 1016I + 1534 C haplotype conferred a stronger survival advantage, highlighting a trade-off between fitness and resistance [108].

Moreover, Uemura et al. investigated the evolutionary dynamics of *Ae. aegypti* carrying the highly resistant L982 W + F1534 C haplotype. Their study revealed that, under laboratory conditions, these mutations rapidly declined in frequency over 15 generations, with only 2.9% of homozygous mutant individuals remaining at the end of the experiment [75]. This suggests a significant fitness cost associated with these alleles, leading to the gradual reversion of resistance in the absence of insecticide selection. The mechanisms driving this decline remain underexplored, but potential factors include reduced mating success, impaired blood-feeding behavior, and overall lower survivability [75].

The evolutionary trajectory of kdr mutations is further influenced by ecological variables and population genetics. Freeman et al. conducted a systematic review of over 170 studies on fitness costs associated with insecticide resistance, finding that 60% of experiments reported a measurable cost, particularly in the form of reduced reproductive success and resistance reversion in the absence of insecticides [109]. However, the magnitude of these costs varies across insecticide classes, with organochlorine-resistant mosquitoes exhibiting fewer fitness trade-offs. Their study emphasized the need for rigorous experimental designs that quantify resistance levels, identify resistance mechanisms, and assess multiple fitness parameters to accurately determine evolutionary trade-offs [109].

Additionally, ffrench-Constant and Bass discussed how fitness costs in resistant insects could be mitigated by compensatory mutations or genetic modifiers, potentially stabilizing resistance alleles in field populations despite inherent disadvantages. They also emphasized that the assumption of a universal fitness cost is oversimplified, as preexisting polymorphisms and complex gene interactions can shape resistance dynamics in unpredictable ways [110].

Taken together, these findings emphasize the importance of considering both laboratory and field data when evaluating the long-term stability of kdr mutations in *Ae. aegypti* populations. Resistance management strategies should integrate this knowledge, as intermittent insecticide use may contribute to the natural decline of kdr alleles due to fitness costs, whereas continuous selection pressure may drive the fixation of highly resistant haplotypes. Future research on how different resistance mechanisms interact with environmental factors will be critical for predicting resistance trends and optimizing vector control strategies.

## Evolution of methodologies for identifying kdr mutations in *Aedes aegypti*

Over the past few decades, methodological advances have significantly improved our ability to detect and characterize kdr mutations in *Ae. aegypti* populations [30], thereby enhancing our understanding of pyrethroid resistance and informing the development of effective vector control strategies. This section explores the evolution of these methodologies, their impact on resistance mapping, and the challenges that remain [111].

### PCR and Sanger sequencing

The identification of kdr mutations initially relied on PCR-based methods, such as allele-specific PCR (AS-PCR) and Sanger sequencing. PCR-based methods provided precise detection of well-characterized mutations such as V1016G/I and F1534 C in the *Vgsc* gene, but they have scalability limitations for the simultaneous detection of multiple mutations [66]. Although AS-PCR is widely used owing to its low cost and low error rate, its applicability may be restricted in large-scale surveillance studies, where a high number of samples must be processed in a short time and where kdr resistance dynamics fluctuate over short periods [66]. High-performance methods such as real-time PCR with TaqMan probes and high-resolution melting (HRM) analysis offer greater sensitivity and speed, but their high cost and the need for specialized equipment may limit their implementation in resource-limited laboratories [66]. In this context, multiplex PCR has emerged as an effective alternative, enabling the simultaneous detection of multiple known kdr mutations in a single reaction, optimizing both monitoring time and costs in resistance management programs [66].

Sanger sequencing enables precise analysis of the *Vgsc* gene by sequencing the affected domains. This method facilitates the precise identification of single-nucleotide polymorphisms (SNPs) associated with resistance, enabling deeper understanding of how specific genetic changes correlated with phenotypic resistance [31, 66, 82, 112]. A significant advantage of Sanger sequencing is that, by amplifying commonly studied regions, such as the domain II S6 segment, it becomes possible to detect multiple mutations within a single assay [113, 114]. This capability extends the utility of Sanger sequencing beyond a limited set of mutations, making it suitable for broader mutation screening within known regions [30, 66, 82]. However, this method remains limited in scalability, particularly for detecting low-frequency mutations in large sample sizes, which also increases costs for high-volume analyses.

### High-throughput genotyping and real-time PCR

As the field has progressed, high-throughput genotyping methods have emerged, allowing for the rapid screening of large populations for multiple kdr mutations. Techniques such as real-time PCR (qPCR) have been widely adopted and allow for the quantification of specific mutations in *Ae. aegypti* populations [21, 84, 87, 114, 115]. qPCR offers several advantages over end-point PCR, including increased sensitivity, the ability to detect low-frequency mutations, and the capacity for quantitative analysis, marking a significant improvement in large-scale resistance monitoring. The development of advanced techniques such as quantitative polymerase chain reaction coupled with high-resolution melting analysis (qPCR-HRMA) has further improved the detection and efficient quantification of kdr mutations in *Ae. aegypti* [26, 28, 104].

These advances have allowed researchers to generate more comprehensive data on the global distribution of resistance alleles, providing a clearer picture of how kdr mutations spread. However, these methods still focus primarily on known mutations, limiting their ability to uncover novel resistance mechanisms. This gap in understanding poses a challenge to predicting how resistance may evolve in response to selective pressures, particularly in regions where resistance is newly emerging.

### Next-generation sequencing (NGS) and genomic approaches

Next-generation sequencing (NGS) allows the sequencing of entire genomes or targeted regions at unprecedented depth and scale. The advent of NGS marked a significant leap forward in the study of kdr mutations, providing much more detailed understanding of the genetic basis of pyrethroid resistance [30, 69, 116, 117]. NGS has revolutionized the DNA sequencing field and enabled the discovery of novel mutations that may contribute to resistance, beyond the well-characterized kdr mutations [118, 119]. This is critical for identifying emerging resistance mechanisms that may compromise the effectiveness of existing control strategies [69, 75]. However, further biological studies, both in the field and in the laboratory, are still needed to establish the correlation between NGS results and insecticide resistance.

NGS also allows for the simultaneous analysis of multiple resistance mechanisms, including metabolic resistance pathways, providing a more holistic view of the resistance landscape [120]. In addition, NGS facilitates the study of genetic diversity within and between mosquito populations, helping to trace the evolutionary origins and spread of resistance alleles. This has important implications for understanding how resistance evolves in response to selective pressures and for predicting future trends in resistance [69, 75].

However, the complexity and volume of data generated by NGS presents challenges in data management and interpretation. Integrating NGS data with other types of data (e.g., environmental, phenotypic) requires advanced bioinformatics tools and expertise, highlighting the need for interdisciplinary collaboration in resistance research.

### Advances in bioinformatics and data integration

The vast amount of data generated by NGS and other high-throughput methods has necessitated the development of advanced bioinformatics tools for data analysis and interpretation [119]. Bioinformatics enables the integration of genomic, transcriptomic, and phenotypic data, comprehensive view of resistance patterns and their association with environmental, usage, and population dynamics factors in other organisms [121, 122]. A notable example is the use of targeted sequencing approaches, such as hybridization capture probe sets, which facilitate the enrichment of specific genomic regions, such as the *Vgsc* gene in *Ae. albopictus*, *Ae. aegypti*, and *Culex pipiens* complex [119]. The development of the automated pipeline presented by Itokawa et al., MoNaS, further streamlines the identification of resistance-associated mutations, enhancing the efficiency of downstream analyses [119]. However, the complexity of these data integration requires careful standardization and validation to ensure that the resulting insights are robust and applicable across different contexts.

### Challenges and future directions

Notwithstanding the aforementioned methodological advances, a number of challenges remain in the mapping of kdr mutations. A significant challenge is the necessity for the implementation of more standardized protocols across studies, to ensure the comparability of the resulting data. The inconsistency in methodologies employed for the detection and characterization of kdr mutations may result in discrepancies in data reporting and interpretation, thereby hindering efforts to establish an integrated understanding of resistance patterns [123–125].

A significant challenge is the lack of standardization in the reporting methodologies for sequencing and resistance data. This variability hampers the capacity to perform comprehensive analyses and to compare sequencing results across different studies, regions, or countries. The implementation of standardized reporting protocols and databases would enhance the capacity to connect and compare findings across regions and time periods, facilitating more accurate global resistance mapping and enabling collaborative efforts in resistance management [123, 125].

A further challenge is the cost and accessibility of advanced genomic technologies. Although NGS and other high-throughput methods have revolutionized the field, they remain costly and require specialized expertise and equipment [126, 127]. This can limit their use in resource-limited settings, where pyrethroid resistance is frequently a significant concern [125]. Addressing this challenge will require efforts to make these technologies more affordable and accessible, possibly through the development of simplified, cost-effective sequencing platforms or through international collaborations that provide technical and financial support to regions with limited resources [123].

In the future, research should concentrate on improving the cost-effectiveness and accessibility of advanced genomic tools, as well as on developing new methodologies for detecting and characterizing resistance in real time. There is also a need for more longitudinal studies that monitor the evolution of kdr mutations over time and across different ecological contexts, which will provide the data essential for developing dynamic and adaptive resistance management strategies [128]. These studies will be pivotal for elucidating the mechanisms underlying the development and persistence of resistance, as well as for assessing the long-term efficacy of diverse vector control strategies.

## Remarks and implications on pyrethroids use

The continued reliance on pyrethroids as a primary chemical vector control measure, despite the alarming global increase in resistance and the widespread proliferation of kdr mutations in *Ae. aegypti*, reflects the complex interplay of practical, historical, and strategic considerations. These factors not only explain the sustained reliance on pyrethroids but also highlight the challenges and rationale behind their ongoing use [129, 130].

### Proven initial efficacy

Since their introduction, pyrethroids have demonstrated unparalleled efficacy in controlling mosquito populations, characterized by their high lethality to mosquitoes and relatively low toxicity to humans (and mammals) [131]. This favorable cost–benefit ratio has established pyrethroids as a fundamental element of vector control strategies, particularly in regions where alternative interventions may be economically or logistically prohibitive [132]. The substantial reduction in mosquito-borne disease transmission initially achieved by pyrethroid use has continued to justify their widespread adoption in many areas, even as resistance has emerged [131, 132].

### Rapid action and versatility

One of the most compelling advantages of pyrethroids is their rapid mode of action. Upon exposure, mosquitoes experience immediate paralysis and subsequent death, often within minutes. This rapid response is particularly valuable in emergency situations, such as during outbreaks of dengue, Zika, and chikungunya, where there is an urgent need to suppress mosquito populations and reduce disease transmission. In addition, pyrethroids offer versatility in applications and are effective for treating bed nets, space spraying, and residual treatments. This adaptability enhances their role in comprehensive vector control programs and provides public health officials with flexibility in diverse operational contexts [52, 131, 132].

### Limited alternatives

Although the rise of pyrethroid resistance is a growing concern, the alternatives—such as organophosphates, carbamates, and neonicotinoids—each present their own challenges. Some alternatives may pose greater risks to human health or the environment [118, 133], while others may be more expensive or less available in certain regions [134]. In addition, the process of developing new classes of insecticides is both time-consuming and expensive, resulting in a limited arsenal of viable chemical options [135]. As a result, public health officials often have no choice but to continue to rely on pyrethroids, despite the challenges posed by resistance [100, 134].

### Resistance management strategies

In many regions, vector control programs have adapted to the challenges of pyrethroid resistance by incorporating these insecticides into integrated resistance management strategies. Rather than relying solely on pyrethroids, these strategies include rotating insecticides with different modes of action to delay resistance development, utilizing mixtures of different chemical classes, and implementing integrated vector management (IVM) approaches [44, 54]. Additionally, IVM combines chemical control with biological control methods, such as the release of sterile insects or the introduction of natural predators, and emphasizes source reduction to eliminate mosquito breeding sites. With careful management and continuous monitoring, pyrethroids can remain as an effective tool in many regions, even in the face of increasing resistance.

### Widespread access and acceptance

Pyrethroid insecticides are broadly accepted by both communities and public health professionals, partially owing to their long-standing use in vector control programs. Transitioning to alternative methods or products would require significant efforts in education, supply chain adjustments, and the adaptation of existing practices, all of which may be challenging to implement, especially in resource-limited settings [134, 136]. The established infrastructure for the distribution and application of pyrethroids ensures that they remain a practical and reliable choice for many vector control programs worldwide.

### Ongoing efficacy in certain regions

Despite the increasing prevalence of resistance, pyrethroids have not been universally undermined. In some regions, these insecticides continue to provide effective control of mosquito populations, particularly when applied correctly and in conjunction with other interventions [51, 137]. The variability of resistance levels in different geographical areas indicates that pyrethroids can still be useful in specific contexts, where they help to reduce mosquito populations and, consequently, the transmission of vector-borne diseases.

## Gaps in current research and future directions

Despite the significant progress made in identifying and mapping kdr mutations, several critical gaps remain in our understanding, particularly regarding how these mutations interact with other resistance mechanisms and their broader implications for vector control. Addressing these gaps is essential for developing comprehensive and effective resistance management strategies.

### Interaction between kdr mutations and metabolic resistance mechanisms

An important area of promising investigation is the interaction between kdr mutations and metabolic resistance mechanisms, such as the upregulation of detoxification enzymes including cytochrome P450 s, glutathione *S*-transferases (GSTs), and esterases [33, 37–40]. Studies have suggested that these interactions enhance resistance levels, thus complicating control efforts. For example, the co-occurrence of VGSC mutations with overexpressed P450 enzymes has been shown to result in significantly higher resistance rates [138, 139]. Understanding these interactions is critical for developing integrated resistance management strategies that address multiple resistance simultaneously [140].

### Geographic variability and region-specific research needs

There is also a significant gap in our understanding of the geographic variability in the prevalence of kdr mutations and the factors that drive this variability. While global studies have mapped the distribution of major kdr mutations, the ecological and genetic factors that influence their spread remain poorly understood [114, 141]. Regional variations in insecticide use, mosquito population dynamics, and environmental conditions all contribute to differences in resistance patterns; however, these factors have not been studied comprehensively. In addition, there is a need for more region-specific studies, particularly in understudied areas such as rural regions and certain parts of Africa and Southeast Asia. These areas may harbor unique resistance profiles that could affect the overall success of global vector control efforts. Expanding research efforts to these understudied regions will provide a more complete picture of the global resistance landscape and inform more targeted and effective control efforts [142].

### Challenges in correlating kdr mutations with specific pyrethroid use

Although significant progress has been made in understanding the correlation between kdr mutation frequency and specific pyrethroid use patterns, several gaps remain. One critical issue is the need for more comprehensive and standardized data on the use of specific pyrethroids, particularly in regions where resistance is emerging. Improved data collection and reporting practices would enable more accurate assessments of the relationship between specific insecticide application and resistance development [143, 144]. Furthermore, the interaction between kdr mutations and other forms of resistance, such as metabolic resistance, in the context of specific pyrethroid use requires further exploration. Understanding how these different mechanisms interact with specific pyrethroids is crucial for developing more effective resistance management strategies [145]. Additionally, more research is needed on the long-term impacts of different insecticide use patterns, including the potential for resistance to revert if selection pressure from specific pyrethroids is reduced.

### Variability in the reporting of kdr mutations and haplotypes

During the literature review, significant variability was observed in the description and publication of kdr mutations and haplotypes. For instance, some authors report mutations as F1534 C, while others refer to the same mutation simply as 1534 C. This inconsistency extends to the reporting of haplotypes as well. Some studies have described, for example, haplotypes using only the resultant amino acids at different positions, such as PPCC for positions 989 and 1534. Others use different notations such as PP/CC or PP + CC, while still others include the position numbers, such as 989P/1534 C or S989P + F1534 C. This lack of standardization in reporting creates challenges for comparing studies and synthesizing data across different research efforts. It also complicates meta-analyses and the development of a cohesive understanding of resistance patterns. Moving forward, it is crucial to establish standardized reporting guidelines for kdr mutations and haplotypes to improve the clarity and comparability of research findings [30, 48, 49, 72, 73, 146]. Furthermore, the lack of standardization in bioassays and biochemical tests to assess phenotypic resistance and metabolic mechanisms exacerbates these challenges, emphasizing the need for unified protocols that integrate genetic and functional data [66, 144].

### Need for high-resolution structural and functional studies of VGSC

While the structural aspects of the VGSC protein have been studied to some extent, there remains a need for more detailed functional studies that explore how specific mutations affect the biophysical properties of the sodium channel [16, 147, 148]. High-resolution structural models of the VGSC, particularly in its mutated forms, are necessary to fully understand how these mutations impact pyrethroid binding and the overall function of the channel [27, 52, 59]. Such studies could provide deeper insights into the molecular mechanisms of resistance and inform the development of new insecticides or resistance management strategies [147, 149].

## Conclusions

This narrative review highlights the complexities and challenges associated with managing *Ae. aegypti* populations in the face of widespread pyrethroid resistance, driven primarily by kdr mutations in the *Vgsc* gene. The emergence and spread of these mutations in several regions, particularly in Latin America, Southeast Asia, and Africa, emphasizes the significant selective pressure exerted by the extensive use of pyrethroids in vector control programs.

One of the key findings of this review is the diverse landscape of kdr mutations, with variations in their prevalence and impact across different geographic regions. The V1016I and F1534 C mutations have emerged as dominant alleles in many populations, often occurring together and leading to enhanced resistance phenotypes. The identification of additional mutations, such as V410L, G923 V, L982 W, and S989P, further complicates the resistance profile and demonstrates the dynamic nature of resistance evolution in response to insecticide pressure.

This review also highlights the critical role of structural and functional studies of VGSC in understanding the mechanisms underlying resistance. Detailed analysis of *Vgsc* gene mutations has provided valuable insights into how these genetic changes alter the biophysical properties of the sodium channel, thereby reducing the efficacy of pyrethroids. However, significant gaps in our understanding remain, particularly regarding the interaction between kdr mutations and other resistance mechanisms, such as metabolic resistance.

In addition, this review highlights the global efforts to map and monitor kdr mutations, which have revealed important regional differences in resistance patterns. These differences are often related to local insecticide use practices, environmental conditions, and mosquito population dynamics. Despite these efforts, there is a clear need for more standardized methods and comprehensive data collection to ensure the comparability and reliability of resistance data across studies.

Despite rising levels of resistance, the persistence of pyrethroids as a cornerstone of chemical vector control reflects the ongoing challenges in identifying and implementing effective alternatives. Although pyrethroids continue to provide rapid and versatile control in certain contexts, their long-term efficacy is increasingly undermined by the spread of resistance. This situation highlights the importance of integrating chemical control with other strategies, such as biological control and community engagement, to develop more sustainable and effective vector management programs.

Looking forward, future research should focus on addressing the identified gaps, particularly through longitudinal studies that track the evolution of resistance over time, and through the development of new insecticides and resistance management strategies. Furthermore, the establishment of standardized reporting guidelines for kdr mutations and haplotypes is vital for advancing our understanding of resistance mechanisms and for guiding the global response to the growing challenge of insecticide resistance in *Ae. aegypti*.

In conclusion, while significant progress has been made in understanding and managing kdr-based resistance, the complexity and variability of resistance patterns require a multifaceted approach to vector control. The integration of chemical, biological, and community-based strategies, supported by ongoing research and surveillance, will be critical in mitigating the impact of insecticide resistance and in protecting public health from mosquito-borne diseases.

## Data Availability

No datasets were generated or analyzed during the current study.

## Abbreviations

DDT: Dichloro-diphenyl-trichloroethane
DNA: Deoxyribonucleic acid
kdr: Knockdown resistance
VGSC: Voltage-gated sodium channel protein
*Vgsc* gene: Voltage-gated sodium channel gene
IRS: Indoor residual spraying
LLIN: Long-lasting insecticidal nets
PCR: Polymerase chain reaction
AS-PCR: Allele-specific polymerase chain reaction
qPCR: Quantitative polymerase chain reaction
NGS: Next-generation sequencing
IVM: Integrated vector management
DENV: Dengue virus
YFV: Yellow fever virus
